# Modulation of Functional Connectivity in Response to Mirror Visual Feedback in Stroke Survivors: An MEG Study

**DOI:** 10.3390/brainsci11101284

**Published:** 2021-09-28

**Authors:** Ruei-Yi Tai, Jun-Ding Zhu, Chih-Chi Chen, Yu-Wei Hsieh, Chia-Hsiung Cheng

**Affiliations:** 1 Department of Neurology, Taipei Medical University Hospital, Taipei 110, Taiwan; 153079@h.tmu.edu.tw; 2 Taipei Neuroscience Institute, Taipei Medical University, Taipei 110, Taiwan; 3 Institute of Brain Science, National Yang Ming Chiao Tung University, Taipei 112, Taiwan; junding0818.y@nycu.edu.tw; 4 Department of Physical Medicine and Rehabilitation, Chang Gung Memorial Hospital, Linkou 333, Taiwan; m7041@cgmh.org.tw; 5 School of Medicine, College of Medicine, Chang Gung University, Taoyuan 333, Taiwan; 6 Department of Occupational Therapy and Graduate Institute of Behavioral Sciences, College of Medicine, Chang Gung University, Taoyuan 333, Taiwan; 7 Healthy Aging Research Center, Chang Gung University, Taoyuan 333, Taiwan; 8 Department of Psychiatry, Chang Gung Memorial Hospital, Linkou 333, Taiwan

**Keywords:** coherence, functional connectivity, magnetoencephalography (MEG), mirror therapy (MT), mirror visual feedback (MVF), stroke

## Abstract

Background. Several brain regions are activated in response to mirror visual feedback (MVF). However, less is known about how these brain areas and their connectivity are modulated in stroke patients. This study aimed to explore the effects of MVF on brain functional connectivity in stroke patients. Materials and Methods. We enrolled 15 stroke patients who executed Bilateral-No mirror, Bilateral-Mirror, and Unilateral-Mirror conditions. The coherence values among five brain regions of interest in four different frequency bands were calculated from magnetoencephalographic signals. We examined the differences in functional connectivity of each two brain areas between the Bilateral-No mirror and Bilateral-Mirror conditions and between the Bilateral-Mirror and Unilateral-Mirror conditions. Results. The functional connectivity analyses revealed significantly stronger connectivity between the posterior cingulate cortex and primary motor cortex in the beta band (adjusted *p* = 0.04) and possibly stronger connectivity between the precuneus and primary visual cortex in the theta band (adjusted *p* = 0.08) in the Bilateral-Mirror condition than those in the Bilateral-No mirror condition. However, the comparisons between the Bilateral-Mirror and Unilateral-Mirror conditions revealed no significant differences in cortical coherence in all frequency bands. Conclusions. Providing MVF to stroke patients may modulate the lesioned primary motor cortex through visuospatial and attentional cortical networks.

## 1. Introduction

Upper-limb dysfunction after stroke accounts for the majority of neurological deficits, and it critically influences independence in daily activities in stroke patients [1]. Mirror therapy (MT), a promising method in the rehabilitation field, helps to improve upper-limb dysfunction caused by stroke [2,3,4,5]. MT is basically conducted with bilateral hand movements (i.e., bilateral MT), in which the subjects try to move both hands in a simultaneous way and imagine that the mirror reflection of a moving unaffected hand is the affected hand [6]. For patients with severe deficits of upper-limb function, MT can be performed by moving only the unaffected hand with the same imagination task as in bilateral MT [7]. Both MT protocols have therapeutic benefits for upper-limb motor function in stroke patients [5,6,7].

One major reason why mirror therapy promotes the improvement of motor function in patients is mirror visual feedback (MVF). MVF is believed to modulate cortical activations, especially the primary motor cortex (M1), which is directly related to motor function. Several neurophysiological studies using transcranial magnetic stimulation (TMS) have found that providing MVF during unilateral hand movement induces increased excitability in the human M1 or patient’s lesioned M1 [8,9,10,11,12]. A recent magnetoencephalography (MEG) study investigating the different effects of MVF with unilateral and bilateral hand movements on cortical activity in stroke patients demonstrated increased lesioned M1 activations in both conditions [13]. Moreover, patients with stroke may have a decreased ability to modulate interhemispheric inhibition [14]. The asymmetric cortical activities of M1 between bilateral hemispheres after stroke can be normalized by MVF either with a unimanual task by the unaffected hand [15] or with a bimanual task [16]. These previous studies support the effect of MVF on M1 facilitation. To date, there are three hypotheses accounting for the effect of MVF on motor recovery. First, MVF is thought to enhance motor imagery and is related to the mirror neuron system. The other possible mechanism is that MVF may facilitate the recruitment of the dormant motor pathways. Finally, MVF may increase individual’s attention toward the affected limb, which may activate motor networks [17]. However, how MVF modulates the activation of M1 needs to be further explored.

Previous studies suggested that MVF modulated cortical activities in M1 possibly through enriched and various sensory inputs, including visual and somatosensory inputs [6,18]. In addition, MVF may mediate the perceptuo-motor control process and resolve the perceptual incongruence by increasing the attentional resources, which are associated with conscious awareness of sensory feedback and enhanced monitoring of the movement [17]. Therefore, MVF may facilitate motor performance via increased attention toward the paretic limb [17]. MVF is also regarded as being correlated with the mirror neuron system, which is activated when a person observes an action [17]. Therefore, MVF might modulate cortical activities in areas other than the motor cortex. Numerous neuroimaging studies have demonstrated activation of a number of brain areas outside the motor cortex in response to MVF [18,19,20,21,22]. MVF elicits cortical activation in the visual cortex in both healthy controls and stroke patients [21,22]. As a higher-order visual area linked with the mirror neuron system, the superior temporal gyrus (STG) is also activated by MVF [19]. Michielsen et al. found that MVF during bilateral hand movements activates the precuneus and posterior cingulate cortex (PCC) in stroke patients [20]. In other functional magnetic resonance imaging (fMRI) studies, increased activations in the precuneus [18,21] and some regions of the parietal lobe [18] were observed in response to mirrored feedback. The precuneus is reported to be associated with spatial attention and the integration of visuospatial information [20,23], and the PCC is related to information exchange and cognitive control of behavior [24,25]. Therefore, the greater cortical activations of these two brain regions indicate increased attentional demands during MVF [17].

The past research described above has revealed that several brain regions are involved in the modulation following MVF; however, the neural mechanism underlying the collaborative work of these brain areas and the temporal relationship among these brain regions still need further investigation. MEG provides the advantages of eminently good temporal resolution while preserving fair spatial resolution [26], so it is suitable for identifying the changes of cortical activation and assessing network connectivity. Furthermore, it is more in line with clinical practice because the subject can execute the motor tasks in a sitting position during the MEG recording. A few previous studies that employed MEG to study the effects of MVF on brain regions in healthy individuals revealed changes in cortical activations in the motor and somatosensory cortices [27,28,29,30,31]. A recent MEG study examined the connectivity among brain areas modulated by MVF in healthy controls [32]. The participants were asked to perform bilateral finger movements in symmetry or asymmetry, while they were provided MVF during MEG recordings. The results revealed increased coherence of alpha-band connectivity between the primary visual cortex (V1) and STG and an approached significant effect on the connectivity between the PCC and precuneus in the gamma band under both MVF conditions. These findings indicated that MVF activates networks involving visual perception, motor imagery, and attention [32]. To date, no studies have investigated the changes in functional connectivity under MVF in patients with stroke, so whether the network change following MVF observed in the healthy population would be present in stroke patients is uncertain. Many stroke patients with motor deficits also develop cognitive impairments in, for example, attention or perception [33]. These neurological deficits may cause differences in cortical modulation when MVF is applied to stroke patients. Although recent work [32] has yielded advances in the understanding of the neural networks involved in MVF, the evidence from stroke patients is still limited and unclear. Understanding how MVF alters the neural connectivity in stroke patients may benefit the usage of MT in stroke rehabilitation.

In our prior work, MVF with either unilateral or bilateral hand movements induced a significant percentage change in beta oscillatory activity in stroke patients. Beta oscillatory activity is generated in M1 after the electrical stimulation [34]; its strength decreases immediately after the electrical stimulation and then increases above the pre-stimulus level [27]. During the voluntary movement, the increased stage of beta oscillatory activity strength would be abolished [35,36]. Therefore, the results implied the facilitation of the M1 region in the lesioned hemisphere [13]. In addition, MVF with bilateral hand movements elicited more pronounced facilitation [13]. In this study, we extended the previous knowledge to investigate the underlying neural mechanism regarding how MVF modulates the functional connectivity among brain regions related to attention and perception in stroke patients. First, functional connectivity was compared between bilateral hand movements with and without MVF. We hypothesized that stronger functional connectivity would exist during bilateral hand movements with MVF. Second, we investigated the difference in functional connectivity between unilateral hand and bilateral hand movements under MVF. As more marked activation in the motor cortex was found in our prior work, we supposed that bilateral hand movements under MVF might induce stronger functional connectivity.

## 2. Materials and Methods

### 2.1. Participants

Fifteen right-handed, male stroke patients were recruited from the outpatient departments in northern Taiwan. Handedness was measured by the Edinburgh Handedness Inventory [37]. The majority of these participants were from our previous study [13]. Table 1 shows the characteristics of the study participants. Among these patients, five had a hemorrhagic stroke, and 10 patients had an ischemic stroke. Eleven patients had stroke lesions localized in the right hemisphere, and 4 patients had lesions in the left hemisphere. The mean age of all patients was 49.60 ± 8.89 years, and the duration of illness was 5.07 ± 3.49 months. All included stroke patients had been diagnosed with unilateral stroke 1–11 months before the study. The severity of patients’ upper-limb motor deficits ranged from mild to moderate assessed by the Fugl-Meyer Assessment [38], and thus all the participants were capable of executing the motor tasks. In addition, the patients had no other neurological or psychiatric diseases. All participants provided written informed consent after a detailed explanation of the experimental procedure, which was approved by the Institutional Review Board of Taipei Veterans General Hospital (IRB No. 2016-06-006B).

### 2.2. Experimental Tasks

**Figure 1:**
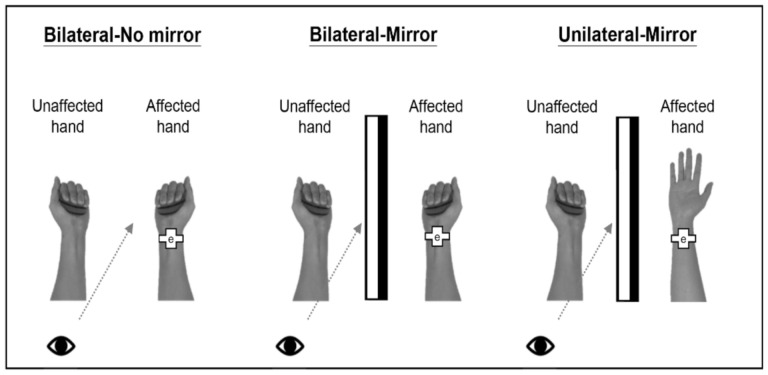
Schematic diagram of 3 experimental conditions. In the Bilateral-No mirror condition, the patients directly observed the affected hand’s movement while performing bilateral hand movements without a mirror. In the Bilateral-Mirror condition, the patients watched the mirror reflection of the unaffected hand’s movement while performing bilateral hand movements. Last, the patients performed the task with only the unaffected hand and observed the mirror image of the unaffected hand’s movement in the Unilateral-Mirror condition. Note: “e” in the pictures indicates the electrode of the electrical stimulator.

This study had 3 experimental conditions (Figure 1) with the order counterbalanced across the patients. The patients performed these 3 tasks consecutively and could rest for 2 min between different experimental conditions. In the first condition, the patients gripped and released a soft ball with the affected and unaffected hands as simultaneously as possible while directly observing the actual movement of the affected hand without MVF (Bilateral-No mirror). In the second condition, the patients performed the same movements as those in the first condition but focused on watching the mirror image of the affected hand’s movement as if it were performed by the unaffected hand (Bilateral-Mirror). The task setting in the third condition was similar to that in the second condition except that the patients gripped and released a ball using the unaffected hand only (Unilateral-Mirror). In both MVF conditions (i.e., the Bilateral-Mirror and Unilateral-Mirror conditions), the affected hand was hidden in a mirror box so that the patients could see the reflection of the movement from the unaffected hand but not the movement of the affected hand. The patients performed the gripping and releasing movements in a rhythmic pattern following the video instructions in all 3 conditions. The camera was set inside the MEG room to monitor the tasks performed by the patients. The researchers observed the patients from the monitor outside the MEG room. Once the patients did not look towards the mirror or dysfunctional limb, the researchers reminded the patients to use the microphone.

### 2.3. MEG Recordings

The patients’ neuromagnetic cortical activities were recorded while the patients executed the 3 experimental conditions inside a whole-head 306-channel MEG (Vectorview, Elekta Neuromag, Helsinki, Finland). Four head indicator coils were used to localize the head position precisely with respect to the sensors. The relative positions between the coil locations and 3 anatomic landmarks of the bilateral pre-auricular points and nasion were decided with a 3D digitizer. Continuing our prior study, which used electrical stimulation to induce beta oscillatory activity for M1 activity analysis [13], we further explored the functional connectivity among the brain regions related to MVF. The electrical stimulation of 0.2 ms constant-current square-wave electrical pulses was delivered to the median nerve of the affected hand with 1.5 s inter-stimulus interval, and supramaximal stimulus intensity was set at 20% above the motor threshold. Here, the electrical stimulation was only used as a mediator to examine the activation of M1. Previous studies have shown that the response of beta rebound induced by electrical stimulation originates in the M1 [27,39], and our previous studies also found that both the voluntary movement and the observation of hand’s movement modulated the M1 activity, which was induced by electrical stimulation through this experimental design [13,40]. The MEG signals from the lesioned hemisphere in each stroke patient were collected, and they were digitized and sampled at 1000 Hz with an online bandpass filter of (0.1, 120) Hz. Each collected MEG epoch for 1000 ms was time-locked to the electrical stimulus onset. The duration of each experimental condition was 4 min, and we recorded more than 90 epochs without artifacts in each condition for subsequent analyses.

In addition, we used surface electromyography (EMG) to monitor patients’ muscle contractions. The EMG patch was attached to the flexor digitorum superficialis of the affected hand for recording muscle twitches, while the patients performed each experimental task. The bandpass filter for muscle signals was set to the range of 20–200 Hz off-line. These EMG signals were then rectified for computing the absolute magnitude and then averaged from the collected artifact-free epochs in each experimental task for quantifying muscle activity over time. The EMG data were captured at a sampling rate of 1000 Hz [41].

### 2.4. MEG Signal Processing and Functional Connectivity Analysis

The temporal signal space separation method was used to eliminate the magnetic interference from the surroundings in the MEG data [42]. The modeling of cortical responses was computed with the Brainstorm software (version: 3 September 2021) [43]. Initially, the noises contaminated by eye blinks were corrected using the signal space projection approach. To solve the problem of forward modeling of MEG measures, an overlapping-sphere head model was applied [44]. The source maps of each patient were geometrically rescaled to the Montreal Neurological Institute brain template (ICBM152). The depth-weighted minimum norm estimate (MNE) was used to compute source cortical activations. Subsequently, the MEG source waveforms of regions of interest (ROIs) were transformed by a Morlet wavelet-based time-frequency approach with the setting of central frequency of 1 Hz and time resolution of 3 s [41]. Each processed MEG epoch of 1000 ms was used for functional connectivity analysis by means of the coherence method in the Brainstorm software. The functional connectivity was determined by estimating source-based coherence among the brain ROIs. Coherence is a statistical measure that computes the relation between two signals, like *x*(*t*) and *y*(*t*), in the frequency domain. The magnitude-squared coherence is,

$$\begin{array}{c} C_{xy}\left(f\right)=\frac{{\left|S_{xy}\left(f\right)\right|}^{2}}{S_{xx}\left(f\right)S_{yy}\left(f\right)}\\ S_{xy}\left(f\right)\;:\;\mathrm{Cross}-\mathrm{spectral}\;\mathrm{density}\\ S_{xx}\left(f\right)\;\mathrm{and}\;S_{xx}\left(f\right)\;:\;\mathrm{Auto}-\mathrm{spectral}\;\mathrm{density} \end{array}$$

The maximum frequency resolution was 1 Hz, and the highest frequency of interest was 50 Hz [32]. Based on a previous review [17] and one recent MEG functional connectivity study [32], we selected the brain ROIs of the M1, PCC, V1, precuneus, and STG in the lesioned hemisphere of each participant. The ROIs in the lesioned hemisphere (right or left) were identified on the Desikan–Killiany template (Table 2).

**Figure 2:**
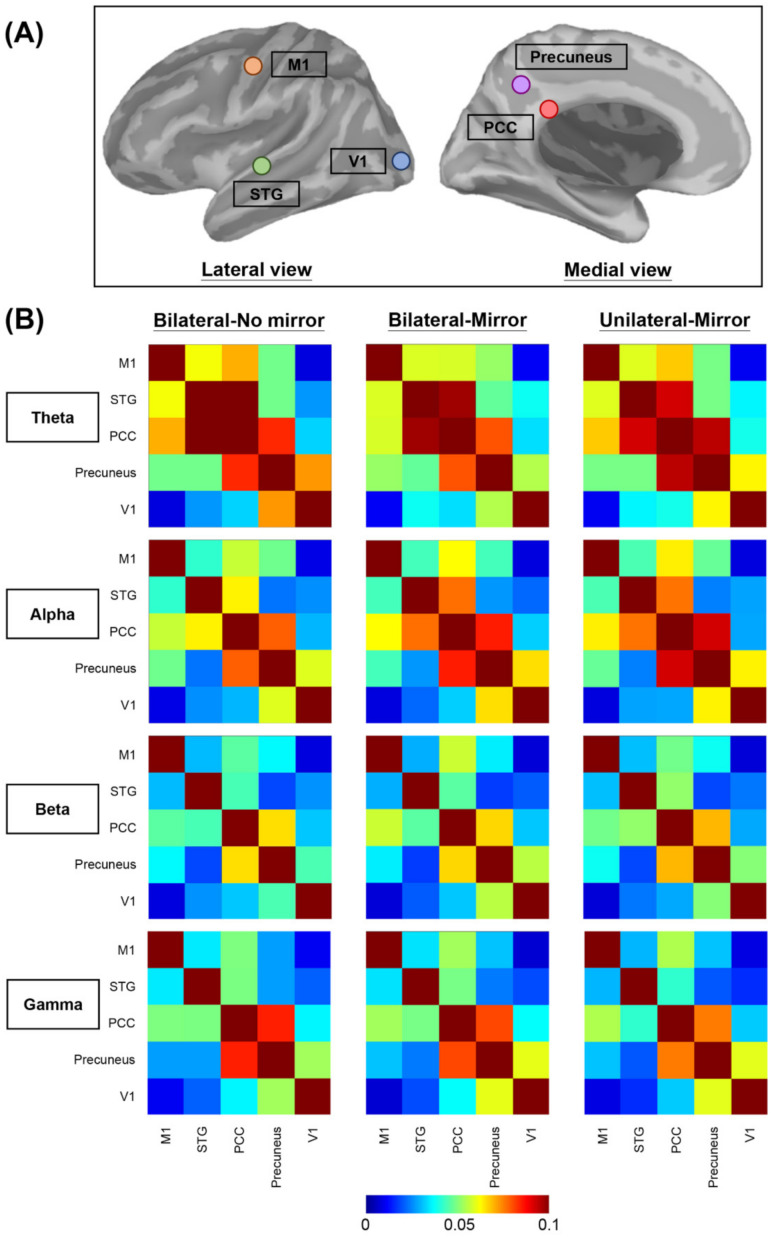
Visualization of the brain connectivity. (**A**) Illustration of 5 brain regions of interest selected for the functional connectivity analysis. (**B**) Averaged functional connectivity matrices among the 5 brain regions of interest in 4 different frequency bands under 3 experimental conditions. Different colors of each grid picture show varying degrees of functional connectivity between each two brain regions. In order to visually present the contrast effect of the relatively small coherence values between different areas in different frequency bands, we presented the color scale using a range of 0–0.1. The original coherence values are demonstrated in the Supplementary Materials. M1—primary motor cortex, PCC—posterior cingulate cortex, STG—superior temporal gyrus, V1—primary visual cortex.

Subsequently, we calculated the coherence values among these areas (Figure 2A). The coherence values were classified and averaged into 4 frequency oscillations (Figure 2B), including θ (5–7 Hz), α (8–12 Hz), β (13–30 Hz), and γ (31–50 Hz) bands [45]. Subsequent statistical analysis was performed using SPSS software.

### 2.5. Statistical Analysis

The differences in functional connectivity between the Bilateral-Mirror and Bilateral-No mirror conditions and those between the Bilateral-Mirror and Unilateral-Mirror conditions were examined by one-tailed nonparametric Wilcoxon signed-rank test. In this study, we selected 5 brain regions and classified the coherence into 4 frequency bands. Therefore, 10 paired comparisons among the 5 brain ROIs in each frequency band were computed. The Benjamini–Hochberg method was applied to adjust the *p* values of 10 comparisons in each frequency band [46].

In addition, we used the Friedman test to compare the differences in EMG activities between the Bilateral-No mirror and Bilateral-Mirror conditions and Bilateral-Mirror and Unilateral-Mirror conditions. The Wilcoxon signed-rank test was applied for post hoc analysis. The adjustment of *p*-values was also computed by Benjamini–Hochberg method. The significant level of the adjusted *p*-value was set at 0.05.

## 3. Results

**Figure 3:**
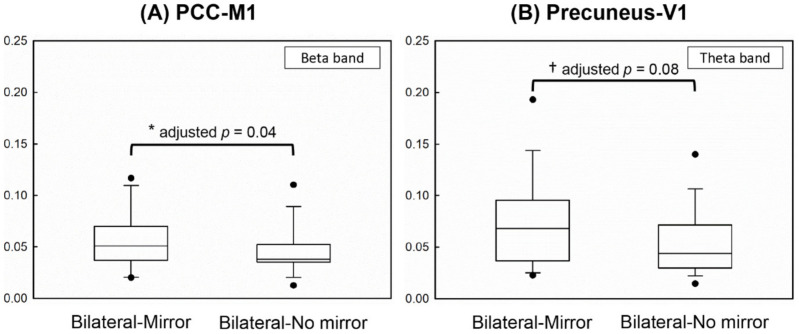
Demonstration of the statistical results using the box plot. (**A**) Significantly higher cortical coherence between PCC and M1 in the beta frequency was found in the Bilateral-Mirror condition than in the Bilateral-No mirror condition. (**B**) The coherence strength between the precuneus and V1 in the theta frequency revealed a greater trend in the Bilateral-Mirror condition than in the Bilateral-No mirror condition. * represents a significant difference after Benjamini–Hochberg method; † represents an approached significant difference.

The results showed that the coherence value between the PCC and M1 in the beta band (adjusted *p* = 0.04) was significantly higher in the Bilateral-Mirror condition than in the Bilateral-No mirror condition (Figure 3A), indicating stronger connectivity between the PCC and M1 in the Bilateral-Mirror condition. In addition, the cortical coherence between the precuneus and V1 in the theta band demonstrated an approached significant difference (adjusted *p* = 0.08), which might suggest higher functional connectivity between the precuneus and V1 in the Bilateral-Mirror condition compared to that in the Bilateral-No mirror condition (Figure 3B).

The detailed comparisons of cortical coherence between any two brain ROIs in the theta, alpha, beta, and gamma bands under the three experimental conditions are respectively presented in Supplementary Tables S1–S4. There was no significant difference in functional connectivity between the Bilateral-Mirror and Bilateral-No mirror conditions in the alpha and gamma frequency bands (Supplementary Tables S2 and S4). In the functional connectivity analyses between the Bilateral-Mirror and Unilateral-Mirror conditions, there were no significant cortical coherence differences among the ROIs in any frequency band (Supplementary Tables S1–S4).

**Figure 4:**
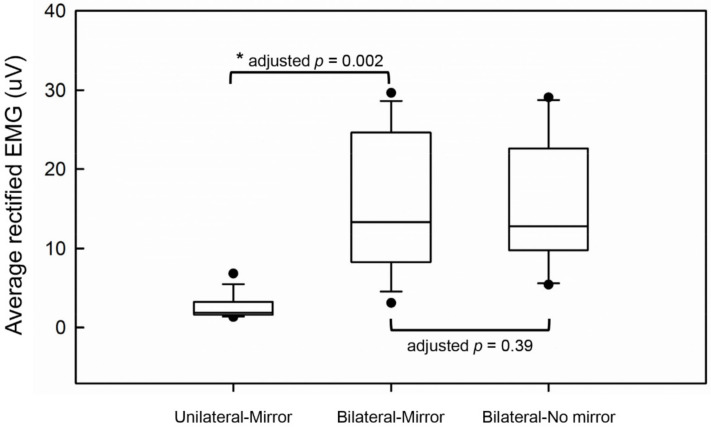
The comparisons of average rectified EMG activities between the experimental conditions. * represents a significant difference.

The comparisons of EMG activities of the affected hand are demonstrated in Figure 4. There was no significant difference in the levels of EMG activities between the Bilateral-No mirror and Bilateral-Mirror conditions (adjusted *p* = 0.39), suggesting the similar motor performance of the affected hand under the two conditions. Moreover, the level of EMG activities under the Bilateral-Mirror condition was significantly higher than that under the Unilateral-Mirror condition (adjusted *p* = 0.002). These results indicated the stroke patients did execute the movement by the affected hand in the conditions with bilateral hand movements.

## 4. Discussion

The aim of our study was to illuminate the possible functional connectivity of some brain areas in the mirror neuron network during either unilateral or bilateral MVF in stroke patients. Therefore, we investigated the differences in functional connectivity among five brain ROIs between Bilateral-Mirror and Bilateral-No mirror conditions and between Bilateral-Mirror and Unilateral-Mirror conditions. Our results revealed stronger cortical coherence between the PCC and M1 in the beta band and possibly higher cortical coherence between the precuneus and V1 in the theta band in the patients performing bilateral hand movements with MVF versus without MVF. Since the motor performance of the affected hand did not differ between the two experimental conditions with bilateral hand movements, these findings indicated the involvement of the attentional network and visuospatial processing in cortical modulation from MVF in stroke patients. However, we found no significant differences in functional connectivity between MVF with bilateral and unilateral hand movements.

Beta oscillatory activity in the M1 is related to motor function, which is suppressed during movements but rebounds after the movements cease [47,48]. In addition, some studies have revealed that MVF during bilateral hand movements can modulate the beta oscillatory activity of the M1 in the lesioned hemisphere in stroke patients [13,16]. The differences in functional connectivity between the PCC and M1 in the beta frequency band with MVF than without MVF during bilateral hand movements suggest that the PCC is functionally associated with the activation of the M1 by MVF. The PCC is highly interconnected with several brain regions, and its function is involved in some part of internally directed thought [49,50]. Beyond this concept, some studies revealed that increased PCC activity associated with improved motor performance may exist in situations requiring externally directed attention [51,52]. Therefore, the PCC is supposed to have a more complex function and to play an active part in balancing internal and external attention [53,54], which might explain the increased functional connectivity in the PCC under our experimental conditions. PCC activity has also been found to increase if a person focuses attention on targets of high motivational value [55]. The stroke patients in our study had strong motivation to improve their motor function and tended to expect better rehabilitation effects during the bilateral hand movements with MVF, which caused them to increase their attention to the MVF. Taken together, MVF might promote M1 activation in stroke patients through the attentional network.

The possibly higher cortical coherence between the precuneus and V1 in the theta band during bilateral hand movements with MVF versus without MVF suggests that MVF might activate the neural network involving visual perception and visuospatial attention. Several previous studies have pointed out that the precuneus is activated during spatially demanding exercises, whether practically executed or imagined [56,57]. In addition, greater activation of the precuneus was observed during a bimanual task requiring complex spatial coordination as compared to one with unimanual movement [23]. In performing bilateral hand movements with MVF, the patients had to move the affected and unaffected hands as simultaneously as possible and imagine that the movement of the unaffected hand’s reflection was the affected one’s. This circumstance made the patient seem to perform the bimanually coordinated movements in an imagined way. Thus, the modulation of V1-precuneus connectivity might be attributed to the integrated effect of MVF and imagery under the condition of bilateral hand movements. Theta waves have been demonstrated to be involved in the organization of visuospatial working memory [58]. Moreover, theta rhythms can be driven by visual stimuli in the visual cortex and modulated according to the level of visual attention [59]. Therefore, the possibly stronger functional connectivity between the precuneus and V1 in the theta band under the condition of bilateral hand movements with MVF may indicate the involvement of additional processing for visuospatial information and attention in the facilitation of the M1. However, this interpretation should be scrutinized because the result only approached significance.

The comparison of unilateral and bilateral hand movements under MVF revealed no significant difference in functional connectivity in spite of the difference in EMG activities. This result implicates the key factor modulating the functional connectivity among the selected brain areas might be the intervention of MVF but not the movement of the affected hand. In our previous study, facilitated M1 activation in the lesioned hemisphere was found either in moving bilateral hands or the unaffected hand only during MVF [13]. These findings suggest that MVF modulates M1 activation via other neural pathways rather than directly activating the M1 because moving only the unaffected hand during MVF could also contribute to M1 activation in the lesioned hemisphere. Moreover, our previous work also demonstrated that executing bilateral hand movements under MVF induced stronger M1 activation than did moving only the unaffected hand under MVF [13]. The difference between bilateral and unilateral hand movements during MVF might suggest an add-on effect through the recruitment of spared motor pathways from the lesioned hemisphere [60] and not the effect of MVF itself. To date, no studies have investigated the functional connectivity among the M1 region and other brain regions under MVF in different motor conditions in stroke patients. The results in this study may provide the information that the role of attentional demand plays in the therapeutic effect of MT, so how to adjust the therapeutic strategy to increase patients’ attention during MT may be a crucial issue in clinical practice. However, this effect should be examined carefully in the future because we only performed a single session of MT, and no control group was included in this study. For more appropriate use of MT in stroke rehabilitation, understanding of the neural mechanism of MT may inspire adjustments of the treatment protocol for stroke patients with heterogeneous neurological deficits. Future study is needed to clarify the effects of MVF under different motor tasks (e.g., uni- or bi-manual tasks) on brain functional connectivity.

Some limitations of this study need to be mentioned. First, the present study recruited 15 individuals with stroke for the analysis. The sample size was relatively modest, and a larger patient group should be enrolled. In addition, a healthy control group should be added. Following the more sophisticated study design, the data analysis should be blinded, and the bias can be eliminated. Second, the stroke characteristics, including the time post-stroke, cortical or subcortical lesions, and ischemic or hemorrhagic type of stroke, may influence the results due to the physiological differences. Subgroup analysis according to different characteristics of stroke should be done in a larger-scale study. Third, we chose the Benjamin-Hochberg method for the adjustment of multiple comparisons due to the relatively small sample size in our study. And, we performed post hoc analysis in each frequency band (i.e., 10 comparisons) separately based on the previous studies [32,61] to examine the significance between any two brain regions in different frequency bands. To acquire more valid statistical results, more stringent multiple testing corrections (e.g., Bonferroni correction) should be used in future studies with larger sample size. Fourth, the coherence method we used to examine the connection between selected brain regions could detect whether the regions networked together or not [62], but it could not provide the directional relationships between the regions. Therefore, the sequence of directions in which the neural network operates is still unclear. Fifth, the number of selected brain regions for the analysis in this study was limited. As in the previous review, brain areas beyond the brain regions selected in the present study were also activated by MVF [17]. More brain areas related to the MVF neural network should be selected for functional connectivity analysis in future research with larger samples. Last, other image modalities, such as fMRI for functional connectivity or diffusion tensor imaging (DTI) for structural connectivity, can be combined in future studies to elucidate the correlation between different brain regions more clearly.

## 5. Conclusions

This is the first neurophysiological study to examine the effects of MVF during different motor tasks on brain functional connectivity in stroke patients. Providing MVF during bilateral hand movements may induce higher functional connectivity between the PCC and M1 in the beta frequency band and possibly stronger connectivity between the precuneus and V1 in the theta frequency band than without MVF. However, no difference in functional connectivity was found between the two mirror conditions. In summary, our results may suggest that MVF may facilitate the activation of M1 in the lesioned hemisphere through visuospatial processing and attentional networks, whether during bilateral or unilateral hand movements. Despite some limitations, our results promote current understanding of the neural networks involved in MVF in stroke patients. These pioneering findings also provide more neurophysiological evidence regarding the effects of MT with bilateral or unilateral movements, which may be useful in adjusting the MT protocol to provide individualized stroke rehabilitation. Future studies with a larger scale and a control group are warranted for proving the implications from this study.

## Figures and Tables

**Table 1:** Clinical characteristics of the stroke subjects.

Sex/Age (Years)	Lesion Location	Stroke Type	Stroke Duration (Months)	FMA-UE
M/38	Right corona radiata	Ischemic	2	60
M/46	Right corona radiata	Ischemic	2	48
M/46	Right MCA	Ischemic	5	60
M/55	Left caudate head	Ischemic	7	56
M/48	Left internal capsule	Ischemic	11	51
M/62	Right corona radiata	Ischemic	6	51
M/63	Right corona radiata	Ischemic	9	41
M/47	Left corona radiata	Ischemic	4	58
M/37	Right MCA	Ischemic	1	55
M/55	Right precentral gyrus	Ischemic	1	60
M/42	Right putamen	Hemorrhagic	4	57
M/62	Right basal ganglion	Hemorrhagic	11	58
M/38	Left basal ganglion	Hemorrhagic	4	37
M/49	Right basal ganglion	Hemorrhagic	8	53
M/56	Right putamen	Hemorrhagic	1	63

Note: Abbreviations: FMA-UE—upper-extremity subscale of Fugl-Meyer Assessment; M—male; MCA—middle cerebral artery.

**Table 2:** The identified ROIs in the lesioned hemisphere on the Desikan-Killiany template.

ROIs	Center of SCS Coordinate	Vertice Number	Area (cm^2^)
Right M1	(20, −47, 95)	353	43.74
Right PCC	(12, −1, 81)	93	12.66
Right V1	(−64, −30, 58)	367	45.57
Right precuneus	(−23, −9, 84)	325	37.95
Right STG	(28, −57, 45)	257	39.58
Left M1	(17, 53, 97)	339	49.58
Left PCC	(10, 1, 80)	85	11.51
Left V1	(−66, 26, 57)	371	45.71
Left precuneus	(−26, 0, 84)	314	36.76
Left STG	(22, 57, 49)	290	43.43

Abbreviations: ROIs—regions of interest; M1—primary motor cortex; PCC—posterior cingulate cortex; V1—primary visual cortex; STG—superior temporal gyrus.

## Data Availability

The data that support the results of this study are available upon reasonable request and can be obtained from the corresponding authors.

## Supplementary Materials

The following are available online at https://www.mdpi.com/article/10.3390/brainsci11101284/s1, Table S1: Cortical coherence between any 2 brain regions of interest in theta band under 3 experimental conditions, Table S2: Cortical coherence between any 2 brain regions of interest in the alpha band under 3 experimental conditions, Table S3: Cortical coherence between any 2 brain regions of interest in the beta band under 3 experimental conditions, Table S4: Cortical coherence between any 2 brain regions of interest in the gamma band under 3 experimental conditions
