# Supplementary material for: Modulation of Functional Connectivity in Response to Mirror Visual Feedback in Stroke Survivors: An MEG Study
Source: Brain Sci. 2021 Sep 28;11(10):1284. doi: 10.3390/brainsci11101284 (PMC8533793; doi:10.3390/brainsci11101284)
Supplement: Supplementary file 1 [file brainsci-11-01284-s001.zip › brainsci-1362916-supplementary.pdf]

# Modulation of Functional Connectivity in Response to Mirror Visual Feedback in Stroke Survivors: An MEG Study

**Authors:** Ruei-Yi Tai, MD, Jun-Ding Zhu, MS, Chih-Chi Chen, MD, Yu-Wei Hsieh, PhD*, Chia-Hsiung Cheng, PhD*

## Supplementary material

**Supplementary Table S1.** Cortical coherence between any 2 brain regions of interest in theta band under 3 experimental conditions

| **Conditions** | M1 _V1 | STG _V1 | PCC _V1 | Precuneus _V1 | STG _M1 | PCC _M1 | Precuneus _M1 | PCC _STG | Precuneus _STG | Precuneus _PCC |
|---|---|---|---|---|---|---|---|---|---|---|
| Bilateral-No mirror | | | | | | | | | | |
| Median | 0.009 | 0.030 | 0.021 | 0.044 | 0.040 | 0.046 | 0.034 | 0.068 | 0.035 | 0.067 |
| IQR | 0.011 | 0.040 | 0.027 | 0.038 | 0.035 | 0.021 | 0.045 | 0.068 | 0.050 | 0.042 |
| Bilateral-Mirror | | | | | | | | | | |
| Median | 0.008 | 0.024 | 0.023 | 0.068 | 0.036 | 0.053 | 0.033 | 0.067 | 0.039 | 0.084 |
| IQR | 0.009 | 0.014 | 0.023 | 0.057 | 0.022 | 0.051 | 0.035 | 0.068 | 0.047 | 0.052 |
| Unilateral-Mirror | | | | | | | | | | |
| Median | 0.010 | 0.026 | 0.038 | 0.054 | 0.041 | 0.055 | 0.042 | 0.064 | 0.037 | 0.082 |
| IQR | 0.011 | 0.047 | 0.044 | 0.039 | 0.039 | 0.032 | 0.037 | 0.044 | 0.029 | 0.057 |
| **Adjusted P value** | | | | | | | | | | |
| Bilateral-No mirror vs. Bilateral-Mirror | 0.46 | 0.15 | 0.54 | 0.08† | 0.50 | 0.13 | 0.49 | 0.50 | 0.54 | 0.11 |
| Bilateral-Mirror vs. Unilateral-Mirror | 0.57 | 0.62 | 0.53 | 0.70 | 0.48 | 0.65 | 0.52 | 0.47 | 0.50 | 0.60 |

The coherence strength is demonstrated by values of median and IQR. The results of the comparison of cortical coherence between different experimental conditions are demonstrated by adjusted p value. † represents an approached significant difference

IQR: interquartile range, M1: primary motor cortex, PCC: posterior cingulate cortex, STG: superior temporal gyrus, V1: primary visual cortex

**Supplementary Table S2.** Cortical coherence between any 2 brain regions of interest in the alpha band under 3 experimental conditions

| Conditions | M1 _V1 | STG _V1 | PCC _V1 | Precuneus _V1 | STG _M1 | PCC _M1 | Precuneus _M1 | PCC _STG | Precuneus _STG | Precuneus _PCC |
|---|---|---|---|---|---|---|---|---|---|---|
| Bilateral-No mirror | | | | | | | | | | |
| Median | 0.007 | 0.014 | 0.025 | 0.062 | 0.035 | 0.052 | 0.031 | 0.046 | 0.020 | 0.073 |
| IQR | 0.011 | 0.025 | 0.016 | 0.051 | 0.021 | 0.031 | 0.053 | 0.039 | 0.017 | 0.043 |
| Bilateral-Mirror | | | | | | | | | | |
| Median | 0.006 | 0.014 | 0.016 | 0.067 | 0.037 | 0.053 | 0.025 | 0.055 | 0.019 | 0.070 |
| IQR | 0.009 | 0.018 | 0.029 | 0.042 | 0.027 | 0.030 | 0.033 | 0.024 | 0.031 | 0.045 |
| Unilateral-Mirror | | | | | | | | | | |
| Median | 0.005 | 0.015 | 0.027 | 0.062 | 0.035 | 0.059 | 0.037 | 0.059 | 0.021 | 0.087 |
| IQR | 0.014 | 0.041 | 0.017 | 0.063 | 0.041 | 0.025 | 0.034 | 0.026 | 0.021 | 0.027 |
| **Adjusted P value** | | | | | | | | | | |
| Bilateral-No mirror vs. Bilateral-Mirror | 0.38 | 0.50 | 0.38 | 0.19 | 0.43 | 0.35 | 0.39 | 0.23 | 0.28 | 0.32 |
| Bilateral-Mirror vs. Unilateral-Mirror | 0.65 | 0.81 | 0.45 | 1.00 | 0.48 | 0.57 | 1.00 | 0.49 | 0.95 | 0.45 |

The coherence strength is demonstrated by values of median and IQR. The results of the comparison of cortical coherence between different experimental conditions are demonstrated by adjusted p value. There was no significant difference in each comparison.

**Supplementary Table S3.** Cortical coherence between any 2 brain regions of interest in the beta band under 3 experimental conditions

| **Conditions** | M1_V1 | STG_V1 | PCC_V1 | Precuneus_V1 | STG_M1 | PCC_M1 | Precuneus_M1 | PCC_STG | Precuneus_STG | Precuneus_PCC |
|---|---|---|---|---|---|---|---|---|---|---|
| Bilateral-No mirror | | | | | | | | | | |
| Median | 0.008 | 0.023 | 0.025 | 0.037 | 0.024 | 0.038 | 0.026 | 0.041 | 0.015 | 0.066 |
| IQR | 0.005 | 0.023 | 0.030 | 0.028 | 0.024 | 0.013 | 0.024 | 0.018 | 0.014 | 0.023 |
| Bilateral-Mirror | | | | | | | | | | |
| Median | 0.008 | 0.015 | 0.033 | 0.047 | 0.020 | 0.051 | 0.033 | 0.041 | 0.016 | 0.063 |
| IQR | 0.006 | 0.016 | 0.016 | 0.055 | 0.018 | 0.030 | 0.032 | 0.014 | 0.011 | 0.031 |
| Unilateral-Mirror | | | | | | | | | | |
| Median | 0.007 | 0.019 | 0.026 | 0.037 | 0.030 | 0.040 | 0.035 | 0.042 | 0.016 | 0.063 |
| IQR | 0.008 | 0.025 | 0.017 | 0.042 | 0.018 | 0.016 | 0.023 | 0.032 | 0.016 | 0.034 |
| **Adjusted P value** | | | | | | | | | | |
| Bilateral-No mirror vs. Bilateral-Mirror | 0.49 | 0.24 | 0.41 | 0.35 | 0.43 | 0.04* | 0.43 | 0.57 | 0.40 | 0.35 |
| Bilateral-Mirror vs. Unilateral-Mirror | 0.45 | 0.43 | 0.38 | 0.48 | 0.19 | 0.10 | 0.42 | 0.47 | 0.45 | 0.33 |

The coherence strength is demonstrated by values of median and IQR. The results of the comparison of cortical coherence between different experimental conditions are demonstrated by adjusted p value. * represents a significant difference (adjusted $p < 0.05$)

**Supplementary Table S4.** Cortical coherence between any 2 brain regions of interest in the gamma band under 3 experimental conditions

| **Conditions** | M1 _V1 | STG _V1 | PCC _V1 | Precuneus _V1 | STG _M1 | PCC _M1 | Precuneus _M1 | PCC _STG | Precuneus _STG | Precuneus _PCC |
|---|---|---|---|---|---|---|---|---|---|---|
| Bilateral-No mirror | | | | | | | | | | |
| Median | 0.010 | 0.016 | 0.035 | 0.045 | 0.033 | 0.042 | 0.027 | 0.058 | 0.022 | 0.080 |
| IQR | 0.007 | 0.014 | 0.021 | 0.026 | 0.028 | 0.021 | 0.008 | 0.033 | 0.023 | 0.050 |
| Bilateral-Mirror | | | | | | | | | | |
| Median | 0.008 | 0.016 | 0.037 | 0.049 | 0.018 | 0.043 | 0.023 | 0.048 | 0.025 | 0.079 |
| IQR | 0.005 | 0.015 | 0.025 | 0.042 | 0.024 | 0.035 | 0.021 | 0.037 | 0.018 | 0.039 |
| Unilateral-Mirror | | | | | | | | | | |
| Median | 0.008 | 0.015 | 0.032 | 0.046 | 0.017 | 0.044 | 0.025 | 0.037 | 0.016 | 0.078 |
| IQR | 0.004 | 0.015 | 0.013 | 0.049 | 0.027 | 0.018 | 0.028 | 0.033 | 0.011 | 0.043 |
| **Adjusted P value** | | | | | | | | | | |
| Bilateral-No mirror vs. Bilateral-Mirror | 0.49 | 0.55 | 0.48 | 0.52 | 0.95 | 0.71 | 0.45 | 0.48 | 0.57 | 0.60 |
| Bilateral-Mirror vs. Unilateral-Mirror | 0.46 | 0.50 | 0.45 | 0.48 | 0.43 | 0.71 | 0.61 | 0.76 | 1.00 | 0.91 |

The coherence strength is demonstrated by values of median and IQR. The results of the comparison of cortical coherence between different experimental conditions are demonstrated by adjusted p value. There was no significant difference in each comparison.
